# Telemedicine in nursing homes: Insights on the social acceptance and ethical acceptability of telemedical consultations

**DOI:** 10.1177/20552076231213444

**Published:** 2023-11-08

**Authors:** Julia Offermann, Martina Ziefle, Nataliya Sira, Dominik Groß, Optimal@NRW Research Group, Saskia Wilhelmy

**Affiliations:** 1 Chair of Communication Science, Human-Computer Interaction Center, 9165 RWTH Aachen University, Aachen, Germany; 2 Institute for History, Theory and Ethics of Medicine, 9165 RWTH Aachen University, Aachen, Germany; 3 Department for Acute and Emergency Medicine, RWTH University Hospital, Aachen, Germany

**Keywords:** Telemedicine, telemedical consultations, nursing homes, social acceptance, ethical acceptability

## Abstract

**Introduction:**

The increasing number of older adults in need of care, the resulting rise in demand for care services and the shortage of nursing staff are major challenges for society. In these situations, the use of telemedicine seems promising – especially in nursing homes when the focus is on rapid support in acute medical cases. However, in addition to the medical and technical potential, the acceptability and usability of the use of telemedical consultations are crucial for a sustainable implementation and acceptance. Our research aims at a holistic identification of socially and ethically relevant parameters for the evaluation of telemedical consultations in nursing homes.

**Methods:**

Presentation of the empirical approach of an interdisciplinary cooperation that combines social and ethical research perspectives during an entire research project. Qualitative analysis of social and ethical aspects based on an interview study with care personnel (N = 14) who have experiences with telemedical consultations in nursing homes, as an example of this interdisciplinary collaboration and to show first insights.

**Results:**

The results of the interview study show a slightly positive evaluation of the use of telemedical consultations in nursing homes. Six main categories were identified to capture and differentiate ethically and socially relevant perceived benefits and barriers (contact with physicians, general, personnel-related, residents-related, technical, and organizational aspects).

**Conclusion:**

The study results allow initial recommendations for the implementation of telemedicine consultations in nursing homes considering socially and ethically relevant aspects. These recommendations can be used to inform medical and technical experts in the field of telemedicine. In addition, the presentation of the interdisciplinary collaboration shows that the close integration of social and ethical aspects in research enables a holistic dimension of the use of telemedicine.

## Introduction

Demographic change has created massive challenges in caring for older adults and those in need of long-term care, due to increasing demand for care services and staff shortages.^1,2^

In Germany, a quarter of all people in need of long-term care are cared for in professional nursing homes, most of which are characterized by a high level of care dependency.^3^ Most of these facilities are run by non-profit or private organizations where trained nursing professionals provide care for people in need of it. In particular, there is an increased need for nursing care in Germany for people over the age of 80.^4^ Various technical solutions aim to support professional caregivers and residents in nursing homes.^5–7^ One promising approach is the use of telemedicine in nursing homes enabling rapid support and assistance in acute medical situations.^8,9^ Their use can eliminate the need for hospital transport and hospitalization, which often leads to disorientation and deterioration of health.^10,11^ This is precisely where the current Optimal@NRW project comes in, providing telemedical support and assistance to 24 nursing homes in the Aachen region (North Rhine-Westphalia [NRW], Germany^12^). The project integrates technical implementation, medical feasibility, efficacy (i.e., reduction of hospitalizations of geriatric nursing home residents), and health economic aspects. In addition, the social acceptance and ethical defensibility of the use of telemedicine in nursing homes are evaluated and assessed.

Previous research on the social acceptance of telemedical consultations mostly applied conventional acceptance models^13,14^ that are limited to few rather generic factors (i.e., ease of use, performance expectancy, etc.) and omit detailed and specific technology-related and contextual impact parameters. Several studies have examined the perspectives of nursing staff,^13,15^ potential patients,^14^ and older adults^16^ on the acceptance and use of telemedical consultations. To date, telemedical consultations in hospitals^15^ or at home^16^ have been studied, while nursing homes have been disregarded.

There are models and catalogs of criteria for the ethical evaluation of sociotechnical applications in medicine^17,18^ that can be used as analytical tools to make statements about the ethical acceptability of human-machine interaction like telemedical consultations in medicine on an individual and societal level.^19,20^ The increasing technical differentiation in medicine, e.g., through the establishment of new telemedical systems, also leads to an increased consideration of ethical aspects.^21,22^ Research on the use of telemedical consultations tends to address ethical issues peripherally,^23,24^ with a few studies focusing on them.^25,26^ In particular, research on the ethical acceptability of telemedicine consultations used in nursing homes and related stakeholders has received little attention.

The aim of our research in this project is to combine the social and ethical research perspectives of all stakeholders involved in the care of patients in nursing homes. In this way, socially and ethically relevant parameters for the evaluation and perception of telemedical consultations can be identified, which are crucial for a sustainable implementation in nursing homes. This paper presents the empirical approach and first interdisciplinary results based on practical experience with telemedical consultations in nursing homes.

## Methods

This section introduces the overall project course and its study concept. Further, the interdisciplinary collaboration between communication science and medical ethics is described in detail with a focus on the empirical approach for investigating the social acceptance and the ethical acceptability of the use of telemedical consultations in the care of geriatric patients in nursing homes. Finally, the participating nursing homes and specific study participants are described.

### Project course and study concept

The underlying research project Optimal@NRW^12^ represents an intersectoral approach to the acute care and support of geriatric patients based on the implementation of telemedical consultation systems in 25 nursing homes in Aachen, Germany. The main objective of the project is to use telemedicine to i) prevent and reduce inappropriate hospital admissions for ambulatory care-sensitive hospital cases and ii) improve medical care in nursing homes. The project provides an additional option for acute care when the general practitioner is not available in acute and severe health situations of the nursing home residents. For this purpose, 25 nursing homes have been equipped with telemedicine equipment for telemedical consultations, and telemedical physicians based at the RWTH Aachen University Hospital are available around the clock to support the nursing homes if general practitioners are not available in time. In addition, mobile non-physician practice assistants (i.e., medical assistants or nurses with at least basic medical training) can be dispatched to perform delegable medical activities (e.g., changing urinary catheters). Within the project, medical and economic analyses are conducted to evaluate the efficacy of the newly implemented structure and processes. Beyond that, the project is accompanied by perspective from social communication science and medical ethics in order to analyze and meet user-related needs and requirements (see Section “Participating nursing homes and study participants”).

Overall, three phases of the project can be distinguished: before usage, during usage, and after usage. During the “prior/after usage” phases relevant parameters, processes, and structures can be identified and provide the opportunity for medical, economic, social, and ethical analyses and comparisons regarding the use of the telemedical infrastructure over the entire project period. The “during usage” phase follows a cluster-randomized controlled intervention trial in stepped-wedge design, so that gradually more and more of the participating nursing homes start to use the telemedical consultations. This phase of the project will allow for an evaluation of the interactions with the telemedical infrastructure in the participating nursing homes in real rather than a scenario-based or laboratory setting. The entire project, including the individual studies of the various project partners, has been reviewed and approved by the Ethics Committee of the RWTH Aachen, Faculty of Medicine.

### Interdisciplinary empirical approach

This section presents the basic procedure of the interdisciplinary approach adopted throughout the project as well as the interaction of communication science and medical ethics for the empirical investigation of both perspectives. While (social) acceptance refers to the general perception and the resulting potential adoption or rejection of facts, products, innovations, or behaviors,^27^ (ethical) acceptability refers to the ethical-normative justification of technology use and thus the reasonableness of individual and societal consequences of technology.^28^ Although the two constructs are closely intertwined,^20^ it is important to note that consideration of one component does not necessarily satisfy the requirements of the other. For it is possible for something to be factually accepted but forbidden and therefore unjustifiable on ethical grounds, and likewise for a measure to be described as acceptable but not be accepted^29^ (p. 57).

**Figure 1.**
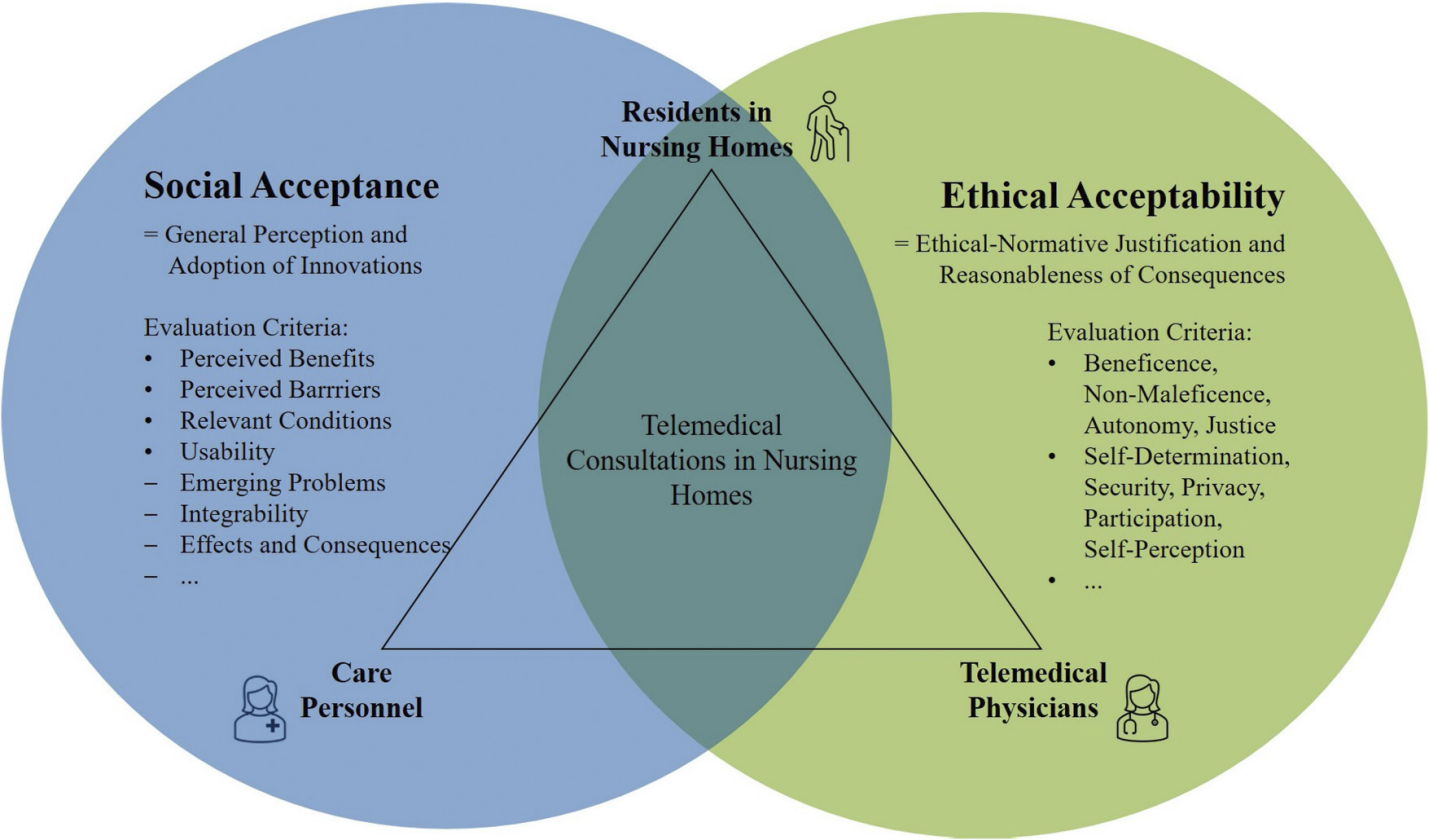
Interweaving and focal points of both interdisciplinary perspectives.

Figure 1 summarizes the social and the ethical perspectives regarding the investigation of telemedical consultations in nursing homes.

From the social perspective, the main focus is on the perspective of the care personnel (in addition to the residents’ perspective), as this group has proven to be the most critical stakeholder regarding the adoption of innovative technologies in professional medical contexts.^30,31^ Here, general perceptions (benefits, barriers, conditions) as well as concrete usability aspects (problems, integrability in and effects on existing structures) are investigated. From the ethical perspective, the focus is predominantly on the residents in the nursing homes and the telemedical physicians (partially extended with the perspective of the care personnel) as well as their mutual interaction. For the ethical analysis, basic ethical principles (beneficence, non-maleficence, autonomy, justice^32^) and ethical criteria (such as self-determination, security, privacy, participation, self-perception^17,18^) regarding the use of technology in medicine are used as relevant bases for evaluation.

**Figure 2.**
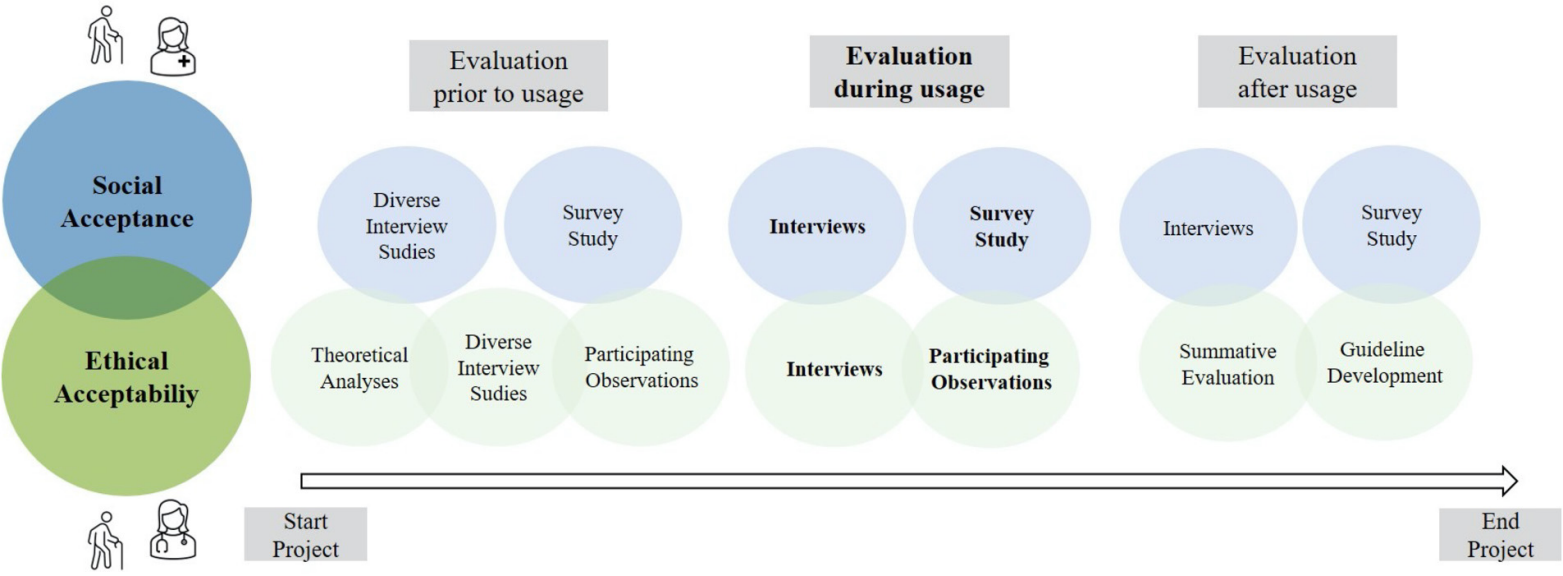
Empirical approach and structure of the collaborative procedure.

Within the underlying research project, a close collaboration between communication science and medical ethics is realized to adequately address all relevant stakeholders and to achieve a holistic evaluation of telemedical consultations in nursing homes regarding their social and ethical dimension. As mentioned before (see Section “Interdisciplinary empirical approach”), the realization of the project follows three phases which were also relevant for the conducted interdisciplinary approach. In each of these phases, different qualitative and quantitative methodological approaches are applied to investigate the acceptance and acceptability of the use of the telemedical consultations in a customized and holistic manner (Figure 2).

For a successful investigation of social acceptance, a series of interview studies with professional caregivers and residents of the participating nursing homes are conducted in the “before usage” phase. Here, the emphasis is on understanding everyday life, including all its challenges and specifics, as well as identifying basic perceptions and attitudes towards the use of innovative approaches such as telemedicine in care.^33^ In addition, the professional caregivers are asked to participate in a survey study to quantify of the identified perceptions and acceptance criteria as a basis for comparisons with later phases of the project. In the phase “during usage”, interviews are conducted with professional caregivers from all participating nursing homes according to the respective cluster assignment, focusing on acceptance, perception, experiences, and usability criteria. An additional quantification by means of surveys is also aimed here. At the end of the project (“after usage”), comparative interview and survey studies are conducted with caregivers from the different nursing homes to enable comparisons with the results of the previous phases of the project.

To investigate the ethical acceptability, theoretical analyses are conducted at the beginning of the “before usage” phase, which will provide an adequate scientific basis for previous ethical findings in the application of telemedical approaches, especially in (professional) care settings. This is combined with a secondary analysis based on the collected results on social acceptance (interview studies) related to ethical criteria. Based on these theoretical findings and participatory observations in the nursing homes, an interview guide is designed. In combination with further participating observations, several interviews are conducted in the nursing homes, focusing on the needs and requirements of the residents. Systematic participating observations and interviews are also conducted in the “during usage” phase: here, the focus is predominantly on the experiences and perceptions of the residents of the nursing homes. In addition, the perceptions, experiences, and evaluations of the telemedical physicians and, in part, the care personnel are examined. Through the lens of these two perspectives, the interaction with the intermediated technology is of particular interest. In the last phase of the project (“after usage”), the results of the different project phases are evaluated and analyzed. Finally, concrete recommendations and guidelines are derived from the identified ethically relevant parameters identified and the determined aspects of social acceptance.

### Participating nursing homes and study participants

25 nursing homes in the region of Aachen in Germany participate in the project and are provided with telemedical equipment. The infrastructure and organization of the nursing homes are very heterogeneous and individual, since the sponsors of the nursing homes differ greatly, e.g., from non-profit organizations, over private organizations leading a large number of nursing homes, to privately funded individual nursing homes. What they all have in common, however, is that the care professionals working in the nursing homes are either specifically trained nursing professionals or nursing assistants. All nursing professionals who participated in any of the project-related studies gave their verbal (interviews) or written (surveys) informed consent to participate.

The nursing home residents are also highly heterogeneous: some homes specialize in caring for residents with advanced dementia, while others have a wide range of residents, from predominantly self-care to those who are permanently bedridden and in need of extensive care (detailed results are already published^33,34^). All nursing home residents gave written informed consent participate in the project and the corresponding interview studies. However, to accommodate the acute conditions and well-being of the residents, additional verbal informed consent was obtained prior to the start of each interview study.

## Results of interview study with professional caregivers

This section presents the approach and results of an exemplary interview study with professional caregivers at the beginning of the “during usage” phase, when six of the 25 nursing homes have started to use the telemedical consultations. First, the methodological procedure is described, followed by the characteristics of the interview participants. Then, the data collection and analysis, as well as the results of the interview study are presented.

### Procedure

All interviews were conducted using semi-structured interview guidelines. In addition, direct interaction with the technology was observed whenever possible. The interviews and observations were conducted by two research assistants of the author group.

The semi-structured interview guideline covered several topics. After a brief introduction to the topic and the underlying research project, participants were asked for *demographic information* such as age, gender, and professional career. Participants then indicated their current position and the length of time they had worked as a professional caregiver in their respective nursing home. The second part of the questionnaire focused on the *participants’ experiences with* and *general perception of the telemedical consultations*. They described situations in which telemedical consultations were used and how they worked. Further, they indicated how they felt when using telemedical consultations and described how they perceived the handling and communication during telemedical consultations in comparison with conventional consultations with on-site physicians. In a third part, participants mentioned, described, and rated various *perceived benefits* of using telemedical consultations. They also described their experiences with *barriers and concerns* related to the use of telemedical consultations. Finally, the participants were asked if and under what circumstances they would like to use telemedical consultations in their future nursing home practice.

### Interview participants

All interviewed participants (N = 14) were professional caregivers working in six different nursing homes belonging to the first cluster of the project study design. There were eight females and six males among the participants. Only care professionals who already had the possibility to use telemedical consultations were included in the interviews: of the 14 care professionals interviewed, 10 led the telemedical consultations and four shadowed. At the time of the interviews (March/April 2022), the participating nursing homes had been using the technology for a few months. The participants were on average 36.1 years old (min = 23, max = 59).

### Data collection and content analysis approach

The interviews were conducted in March/April 2022 at six different nursing homes participating in the research project. All interviews were audio-recorded and transcribed verbatim. The transcripts were anonymized so that no conclusions could be drawn about individuals. Based on the qualitative content analysis approach,^35^ the transcripts were analyzed with the aim of inductively and deductively identifying relevant content, particularly with regard to the perceived benefits and perceived barriers of using telemedical consultations.

### Qualitative results of the interview study

In the following section, interdisciplinary qualitative results from the “during usage” phase are described, starting with insights into the general perception of telemedical consultations in nursing homes, followed by identified motivators and perceived advantages as well as identified barriers to sustainable adoption of telemedical consultations in nursing homes.

#### General perception of telemedical consultations

Summarizing the results, the concept and handling of using telemedical consultations in nursing homes were evaluated slightly positively, with no extremely negative or extremely positive feedback, e.g., “*I felt relaxed and well*” (female, 28 years). When respondents were asked to reflect on the opinions of their colleagues, these were often more negative than their own opinions and were described as skeptical, reserved, or annoyed, e.g., “*The colleagues are currently rather annoyed because of the time that is lost on it*” (male, 23 years).

#### Perceived advantages of telemedical consultations

**Figure 3.**
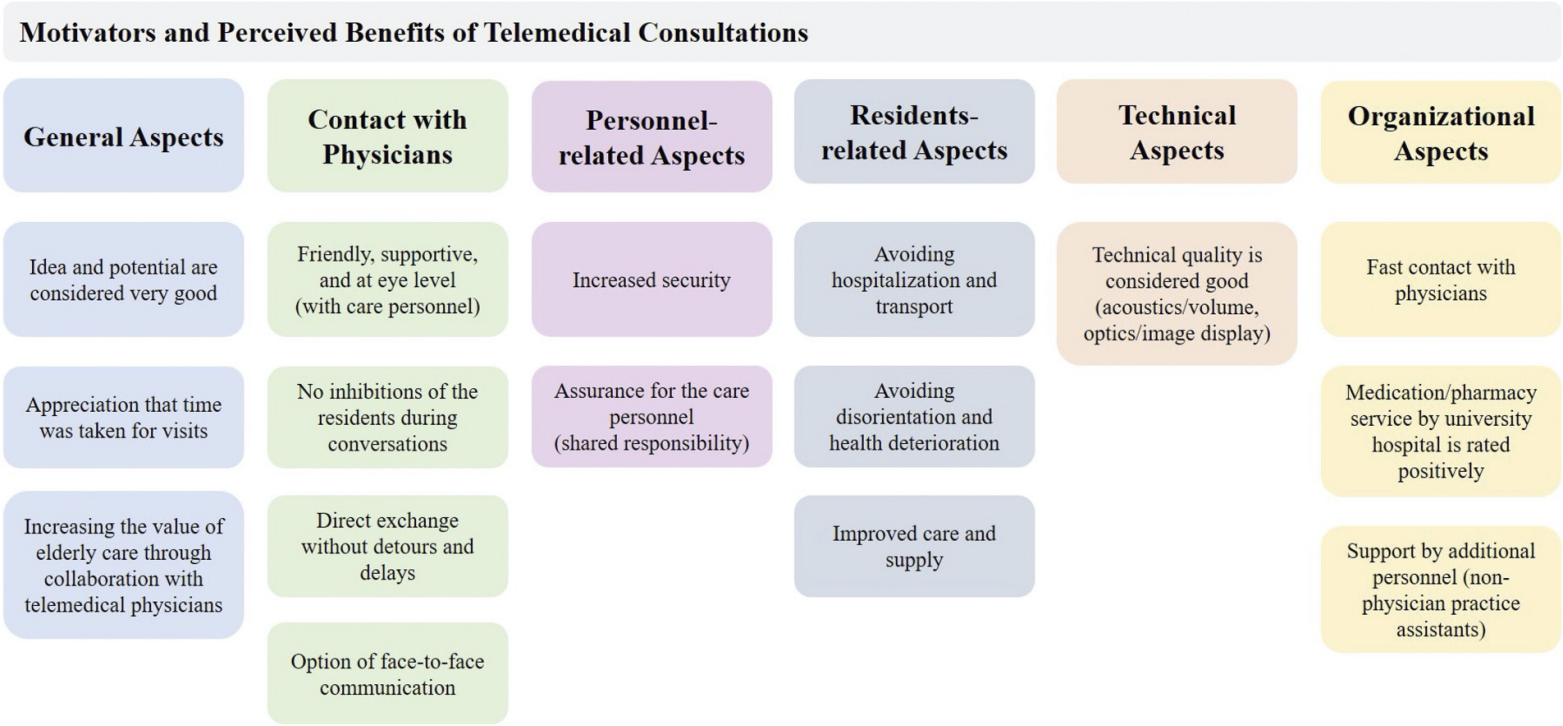
Identified perceived benefits of using telemedical consultations.

Figure 3 shows the identified categories of perceived advantages and motives for the use of telemedical consultations in nursing homes. The results are presented according to the categories identified.

**General Aspects:** Especially the idea and potential of using telemedicine were acknowledged.

*“So, I think the technology is great. (…) It just really helps, and you're secured, and that's the most important thing in our profession.” (female, 31 years)*

In addition, the participants appreciated the time telemedical physicians take for consultations: “*…very friendly and also takes a lot of time”* (male, 23 years). Beyond that, they perceived the collaboration with telemedical physicians as adding value to caring for older adults.

**Contact with Physicians:** Here, most of participants emphasized that the contact was friendly, supportive, and at eye level. Some of the participants described that they did not notice any inhibitions on the part of the nursing home residents during the conversations. In addition, it was acknowledged that the telemedical consultations allowed for a direct exchange without detours or delays, while still allowing for face-to-face communication.

*“You get a printout with the prescriptions, diagnoses, medication directly without having to ask several times as usual…” (male, 25 years)*

*“That I can speak directly to a doctor who can even look at the resident.” (female, 59 years)*

**Personnel-related Aspects:** Most participants described the advantage of increased safety. For example, participants appreciated the ability to protect the nursing staff by sharing responsibility for residents with telemedical physicians.

“*You really feel safer because you are not on your own and you still have a second opinion.” (male, 25 years)*

*“It goes faster, and we are allowed to act, we have to have a doctor's order.” (female, 31 years)*

**Residents-related Aspects:** The benefit of avoiding hospitalization and transportation for nursing home residents was mentioned by almost all participants. Avoiding disorientation and health decline were also relevant benefits of using telemedicine in nursing homes.

*“Then you don't have the drama with the hospitalization unnecessarily, because that's a huge process and it's made easier.” (female, 28 years)*

In addition, participants associated improved resident care and services with the use of telemedicine in their nursing homes.

*“Waiting times are avoided, sometimes you wait for 7 h for the doctor on call, especially in terms of time it is a better care and supply.” (female, 31 years)*

**Technical Aspects:** Participants rated the technical quality of the telemedical system as good and mentioned that adequate acoustics and optics supported communication with the telemedical physicians.

*“There were no problems – acoustically everything was well audible (…) There were no differences to conventional consultations.” (female, 31 years)*

**Organizational Aspects:** The most relevant aspect for the participants was the rapid contact with physicians. This reduced or eliminated enormous waiting times for the medical on-call service.

*“No waiting time for treatments: Teleconsultations are very fast; when I call, someone answers immediately.” (female, 27 years)*

The medication/pharmacy service provided by the university hospital as part of the project was also seen as beneficial because it saved time and reduced the burden on the care personnel.

Finally, the support provided by additional personnel in the form of non-physician practice assistants was also perceived as a major advantage, relief, and support of the care personnel.

#### Perceived barriers and obstacles for a sustainable adoption

**Figure 4.**
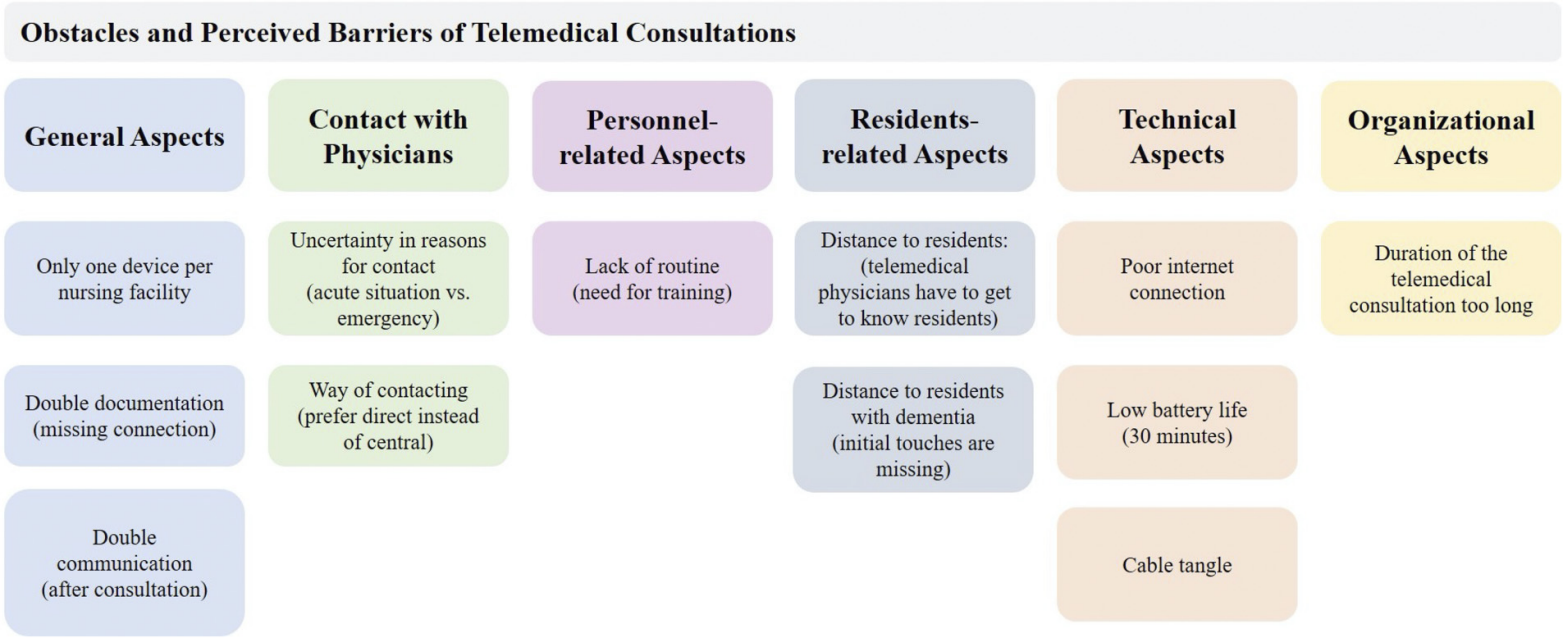
Identified perceived barriers of using telemedical consultations.

During the interviews, participants mentioned several barriers and usability issues they encountered during telemedical consultations in their nursing homes (Figure 4). The barriers and concerns are presented related to the categories identified.

**General Aspects:** The most frequently mentioned problem was that there is only one device for telemedical consultations per nursing home–which poses organizational and infrastructural challenges that are often insurmountable, especially in larger nursing homes (e.g., several floors, sometimes no elevators available at night).

*“I don't know exactly where it is now (…) If I have to look for it, because I don't want to move away from the resident.” (male, 24 years)*

In addition, the current duplication of documentation was criticized. Due to the lack of a direct interface between the central patient file implemented in the project and the different documentation systems of the nursing homes, double documentation is currently necessary: “*I just think, that should be linked together* somehow,“ (male, 24 years). This can lead to errors and put patients at risk, which is a serious ethical issue.

There is also a dual communication channel after consultations: the attending primary care physician is informed about the consultation by both the telemedical physician and the care staff of the respective nursing home.

**Contacting the Physicians:** Here, participants expressed uncertainty regarding the reason for contact, i.e., they were unsure when events and medical conditions were appropriate for telemedical consultation and when they were not.

In addition, care personnel were critical of the way physicians were contacted. Originally, a central call center was supposed to coordinate the access and referrals to the telemedical physicians. During our interviews, it became clear that the participants either did not want to dial the central call center (due to previous experiences in other cases) or that there were technical (i.e., configured direct dialing for telephone systems not possible) and organizational issues (e.g., too long waiting times). Instead, participants preferred a provided direct number to the telemedical physicians’ center and used this way almost exclusively.

“*In any case, the direct number: the problem is, I tried something privately once and it was a disaster. I was forwarded from here to there and in the end, I was later out of the line and had to call again from the beginning.” (female, 59 years)*

**Personnel-related Aspects:** The participants emphasized the lack of routine and the resulting need for training in the regular use of the telemedical consultations.

*“So, until now there's always been one more because I'm… also still a little bit unsure and… don't have the routine in there yet.” (female, 58 years)*

It was noticeable that in some nursing homes, there were a limited number of staff who really used the technology and were happy to do so, while there were also those who were very reluctant and resistant.

**Residents-related Aspects:** Here, the distance to the residents was mentioned. In the interaction during the consultations, it was observed that the telemedical physicians need to get to know the residents, as they do not know the residents’ medical history, unlike the attending primary care physician.

On the other hand, direct physical contact is missing as an important element of communication with residents with dementia:

*“… difficult in the gerontological-psychiatric area: because the residents usually need the initial contact; they need a direct approach…” (female, 31 years)*

**Technical Aspects:** Some participants mentioned a poor internet connection in their nursing homes, short battery life, and cable tangle. The latter was mentioned by several participants and referred to the fact that there were many different cables connected to additional measuring devices of the telemedical system.

*“The only thing that really bothers me is this tangle of cables, I have to be honest.” (female, 31 years)*

**Organizational Aspects:** Another major barrier mentioned by care personnel was organizational and focused on unnecessary examinations. Specifically, several participants described trying to perform non-essential examinations, resulting in consultations that sometimes lasted more than an hour, which was incompatible with the daily care routine.

*“Takes up an incredible amount of time: some rounds have lasted almost 1.5 h (…) and for us in care this is very difficult.” (female, 27 years)*

## Discussion

The key insights of the study are discussed below, followed by recommendations based on the findings and an outlook for future research.

### Key insights

The social and ethical investigations on the new telemedical care system revealed relevant parameters for the use of telemedical consultations in nursing homes. As the evaluation of the systems takes place in iterative cycles, the findings contribute to a better understanding of the system and its use in context. At this stage, both potential advantages and barriers to successful and sustainable adoption have been identified. Beyond previous findings related to telemedical applications used in hospitals or at home,^15,16,26^ this study provided insights into the specific context of nursing homes and the relevant perspectives of nursing home residents and their professional caregivers on the implementation of telemedical consultations in their living environment.

### Social and ethical perspective–derived recommendations

**Figure 5.**
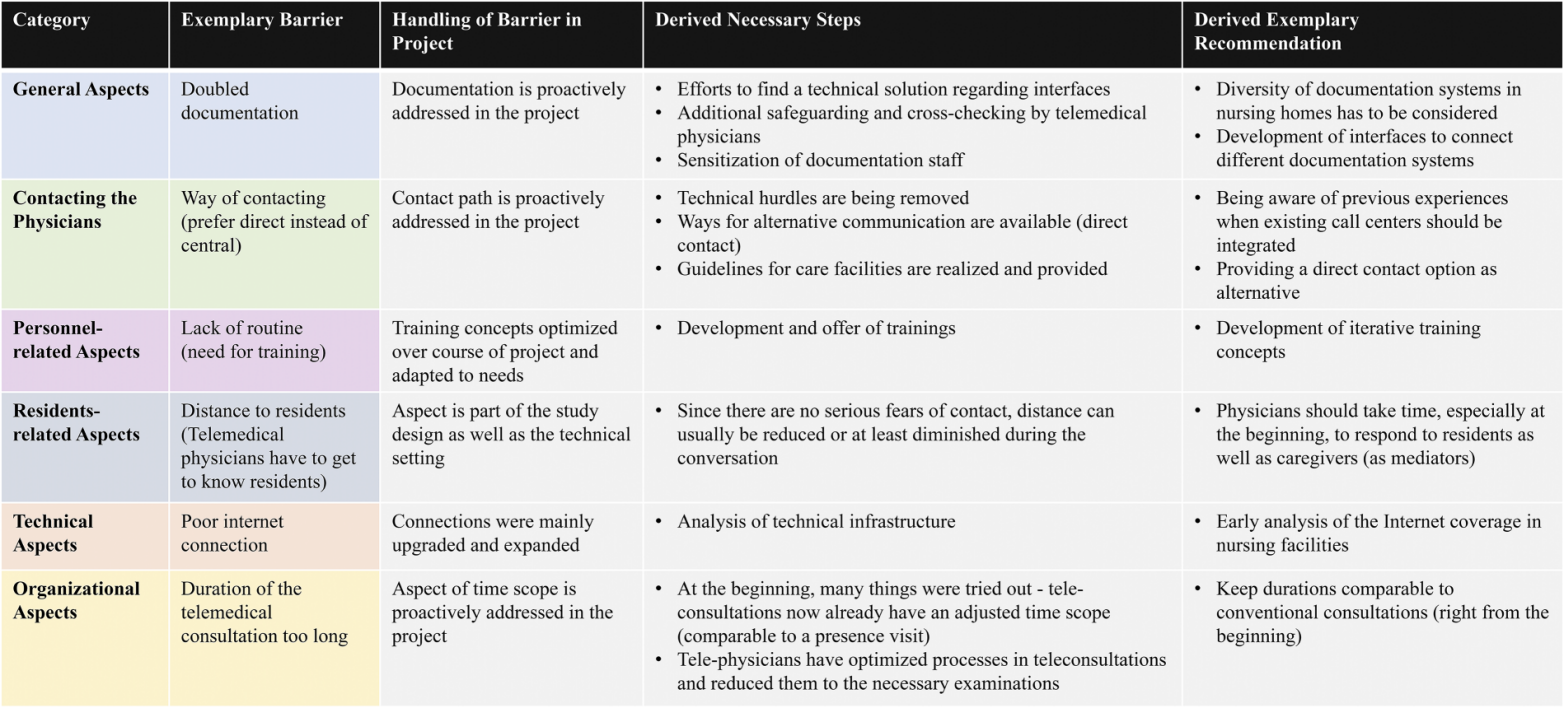
Evaluation of the identified barriers and derived recommendations.

From the initial results of this study, first project-specific recommendations for the implementation of telemedical consultations in nursing homes can be derived, taking into account the social and ethical dimension. Figure 5 shows the derived necessary steps and exemplary recommendations elaborated on some of the identified barriers and their handling during the course of the project.

As an example, the need for *doubled documentation* was identified as an obstacle in the use of telemedical consultations, as it could lead to an increased workload, errors and pose a risk to patients.

From the point of view of social acceptance, the need for doubled documentation plays an essential role for care professionals. The bureaucratic documentation of care is part of a care professional's job, in addition to caring for residents. However, duplicating this task is hardly compatible with the hectic pace of daily work and can be perceived as an additional burden and lead to rejection. In addition, this duplication of documentation can lead to errors in transmission and thus put the patient's health at risk. Risk assessment, i.e., the potential harm to the patient's well-being, is particularly important when considering the ethical acceptability of such documentation.

The identification of this obstacle made it possible to proactively address this issue right at the beginning of the implementation phase (“during usage”). On the one hand, efforts were made to find technical solutions to consolidate the different interfaces (i.e., the documentation software of the nursing homes and the telemedical setup). If technical solutions could not be realized in time, additional safeguarding and cross-checking by the telemedical physicians as well as sensitization of the care personnel were identified as necessary elements within the new intersectoral process. Based on these insights, it is recommended that future projects and implementations of telemedical applications should consider the high heterogeneity in nursing homes, which leads to a wide variety of documentation software and processes in nursing homes. Here, it is of utmost importance to firstly identify and elaborate the individual requirements, infrastructure, and characteristics of the respective nursing home, the care professionals as well as nursing home residents. This should be realized by visiting the nursing homes on site, analyzing the specific requirements on site, and interviewing the respective stakeholders individually. In addition, future research should focus on the development of accessible interfaces that can connect the telemedical application to different documentation systems.

A second example related to the *way of contacting the telemedical physicians*: here, the participating care professionals mostly did not contact the call center that should be contacted to start the intersectoral process; instead, they preferred to contact the telemedical physicians directly. In terms of social acceptance and ethical acceptability, this issue is multidimensional in terms of the stakeholders involved: The fact that the decision as to whether a medical situation was suitable for a telemedicine consultation, a local physician or hospital admission was initially the responsibility of the care professional and not, as planned, of a decision board (call center), led to increasing uncertainty. From a nursing point of view, the direct line to the tele-physician was a reassurance in this decision-making process. From an ethical point of view, the well-being of the patient is relevant in this constellation, which can be endangered if the planned decision-making and responsibility paths do not work, resulting in shifts of responsibility and burdens. Care professionals take on a new role in this responsibility and decision-making process. On the other hand, the viewpoint of the telemedical physicians is also ethically relevant, as they are given additional responsibility in triaging patients and also take up time that they actually need for the care of the patients in the telemedical consultation; here, issues of harm reduction, patient well-being and equity of care all play a role. Immediately after this problem was identified, this issue was also addressed proactively within the project: first, technical barriers to contacting the call center were removed as far as possible, and guidelines for the nursing homes–which should ideally be contacted–were implemented and made available. However, as a back-up it, was very useful in the project to have alternative ways of communication for direct contact with the telemedical physicians available from the beginning, which supported and strengthened the care personnel's trust in the telemedical physicians. Future approaches should take into account that previous experience may play a decisive role when integrating existing call centers into the telemedical infrastructure. Further, a direct contact option should be provided as backup alternative.

Another identified barrier was *the distance to the residents* due to the telemedical way of contact, which meant that the telemedical physicians first had to get to know the residents. Of course, this aspect is part of the study design and technical setting. From an ethical point of view, it is important not to harm patients and to respect their autonomy. Because of the technical distance to the patients, the work of the telemedical physician is a new challenge and sometimes different from that of the patient on site; many of the physical sensations that a physician perceives on site (e.g., palpation, smells) are missing, which are compensated by technical or personnel support (e.g., digital stethoscope, mobile non-physician practice assistants). In this context, telemedical physicians must ensure that the quality of diagnoses and treatments is guaranteed so that patients are not harmed. There is also the aspect of justice, as diagnosis and treatment should be equally available–digitally and on site. Getting to know the patient (anamnesis) is particularly important and is often supported by care professionals who are familiar with the patients and their everyday life. Another aspect of care support in telemedical consultations is the preservation of patient autonomy: Can patients express their autonomy at this distance? Is autonomy compromised by the care professional's interaction with the telemedical physician during a consultation? However, our results showed that there were no serious fears or concerns on the part of the residents. Therefore, the distance was reduced or at least shortened during the telemedical consultation. Future telemedical approaches and attending telemedical physicians should consider the importance of taking time to respond and communicate with residents and their caregivers (sometimes acting as mediators), and of taking them and their needs seriously from the beginning.

The last and probably most relevant aspect for acceptance was the *duration of the telemedical consultations,* which was perceived as too long at the beginning of the project due to partly unnecessary examinations because many things were “tried out”. From an ethical point of view, there are also justice issues. It should be ensured that the time allotted for a telemedical consultation is sufficient to diagnose and treat patients. Exceeding this time leads to inequities compared to other face-to-face patient contacts. It also means that the care professionals involved in the consultation are also time-bound and cannot carry out their busy daily work.

Immediately after this problem was identified, the project proactively addressed and optimized the process: the telemedical physicians adjusted the consultations and their duration to be comparable to conventional face-to-face consultations and reduced the examinations’ time. For this, it should be clarified which examinations are necessary in the medical situation to make a diagnosis and to help the resident. In addition, it should be clarified how procedures can be made more efficient, e.g., the use of the digital stethoscope (guidance of caregivers, preparation of examination materials, technique check). For future telemedical applications and approaches, it is therefore recommended to try to keep the duration comparable to conventional consultations right from the beginning of the implementation phase.

### Conclusion and outlook

The findings are based on a first iteration of the telemedical system (project phase “during usage”), in which some of the participating nursing homes gained first experiences with the use of telemedical consultations. The early integration of social and ethical perspectives on the use of telemedical consultations in nursing homes contributed positively to the further refinement of the system, both in terms of technical and organizational aspects, as well as to the identification of communication issues between different stakeholders in the care context. In the current advanced phase of the project, it is already noticeable that concrete solution strategies are being developed and, as a result, initially existing problems are occurring less frequently (e.g., the duration of telemedical consultations, see section 4.2).

In the further course of the project, pre- and post-comparisons on the ethical and social evaluation of the telemedical consultations will be carried out. Beyond the initial general recommendations presented here, it will eventually be possible to derive concrete ethical and social guidelines at the end of the project, taking into account the needs and requirements of all stakeholders (i.e., residents, professional caregivers, telemedical physicians). The guidelines are intended to ensure compliance with ethical criteria as well as social expectations and requirements of potential users when telemedical applications are to be used in sensitive contexts such as nursing homes.
